# Arsenic in Diseases of the Skin

**Published:** 1844-07

**Authors:** 


*Arsenic in Diseases of the Skin.*—We have to thank Mr. Erichsen for some valuable remarks on the use of arsenic in cutaneous affections. The indiscriminate use of this article in certain diseases of the skin, has no doubt frequently brought discredit upon it.

One great principle to be held in view in its use is never to administer it in the early, acute, and inflammatory stages of the affection. The solution of arsenic of potassa (Fowler's solution) and the solution of the iodide of arsenic and mercury (Donnovan's solution), are the two preparations which are most frequently prescribed in this country; and the former is by far the more popular of the two. Different authors vary a little as to the dose of Fowler's solution which ought to be given; but most of them agree that we ought not to exceed 15, or at most 18 drops in the course of the day. Mr. Erichsen, however, begins with two minims of the solution twice a day, and increases the dose to 5, 6, or 7½ minims three times a day, beyond which, he says, it ought not to be carried, as its good effects will be more evident from small doses continued for some time, than from larger doses which have to be sooner relinquished. Some patients, however, are so excitable that the smallest doses are inadmissible. Another good preparation is the iodide of arsenic, introduced by Dr. A. T. Thompson. The dose is the twelfth of a grain twice a day, to be increased to the sixth or the fourth of a grain three times a day, although these doses are seldom necessary. It is well to combine the extract of conium with iodide of arsenic, in order to sheath its irritating qualities, and prevent it from exciting too powerfully the mucous membrane of the stomach. By the addition of the biniodide of mercury, a compound pill may be formed, which resembles in its effects the liquor of the hydriodate of arsenic and mercury, and which has been found by Dr. A. T. Thompson very efficacious in lupus, and by Mr. Erichsen in some syphilitic eruptions, more particularly when of a squamous kind. A pill may be made containing one-twelfth of a grain of the iodide of arsenic, one-sixth of a grain of the biniodide of mercury, and two grains of the extract of conium; to be given twice a day. The iodide of arsenic may be gradually increased to one-sixth of a grain, and the biniodide of mercury omitted at the end of a fortnight, or sooner, as it might affect the gums. The diluted biniodide of mercury ointment may in some cases, as in syphilitic psoriasis, be also applied externally at the same time. Arsenic seems to be an excitant or stimulating tonic, acting chiefly on the digestive, nervous, and integumentary systems; and when the dose has been pushed so as to irritate any one of these parts, the medicine ought to be immediately suspended, or greatly reduced in strength. The time for prescribing arsenic in a cutaneous affection requires, perhaps, as nice a discrimination as can be exercised. Mr. Erichsen points out a mode of practice which will be generally serviceable in these cases. As it is in the chronic stages that the remedy is to be used, he first ascertains whether the affection will bear the topical application of mild stimulants, such as the ointment of the white precipitate, or of the nitrate of mercury diluted with equal parts of the spermaceti ointment, or a solution of the sulphuret of potassium in the proportion of about a drachm to the pint; and if any of these applications can be borne without increasing permanently the severity of the disease, the internal administration of arsenic may likewise be beneficial. In illustration of this opinion he adduces as an example that very common disease, eczema, which, when, in its acute stage, in which it will bear no other topical application than the most soothing poultices and fomentations, will infallibly be greatly increased in severity by the employment of arsenic, even in its most minute doses; but at a more advanced period, when it can bear topical stimulants, the same disease will be greatly benefitted by the internal administration of this medicine. The diseases of the skin for which arsenic is recommended may be of very different kinds, but they all agree in this, that they are characterized by the presence of scales or scurf; and even when they do not belong to the order *squamæ*, they are usually but little benefitted until they arrive at that stage in which they assume a furfuraceous or scaly condition.—*Braithwaite's Retrospect.*

